# EOLA1 Inhibits Lipopolysaccharide-Induced Vascular Cell Adhesion Molecule-1 Expression by Association with MT2A in ECV304 Cells

**DOI:** 10.1155/2015/301562

**Published:** 2015-12-31

**Authors:** Weiling Leng, Xiaotian Lei, Hao Meng, Xinshou Ouyang, Ziwen Liang

**Affiliations:** ^1^Department of Endocrinology, Southwest Hospital, Third Military Medical University, Chongqing 400038, China; ^2^Section of Digestive Diseases, Department of Internal Medicine, Yale University School of Medicine, New Haven, CT 06519, USA

## Abstract

Our research group firstly discovered endothelial-overexpressed lipopolysaccharide-associated factor 1 (*EOLA1*, GenBank number AY074889) as a lipopolysaccharide (LPS) responsive gene in ECV304 cells. The previous studies have further demonstrated the association of EOLA1 with metallothionein 2A (MT2A), while the role of EOLA1 during LPS-induced inflammatory response in ECV304 cells is unknown. In this report, we determined the subcellular localization of EOLA1 and the regulatory capacity of EOLA1 on vascular cell adhesion molecule-1 (VCAM-1) in response to LPS in ECV304 cells. Our results show that EOLA1 is broadly diffuse in the cells, and EOLA1 expression is dramatically induced by LPS. EOLA1 knockdown results in significant enhancement of LPS-induced VCAM-1 production. Consistent with this, overexpression of EOLA1 leads to the reduction of LPS-induced VCAM-1 production. Furthermore, MT2A knockdown reduces LPS-induced VCAM-1 production. Collectively, our results demonstrate a negative regulatory role of EOLA1 on LPS-induced VCAM-1 expression involving its association with MT2A in ECV304 cells.

## 1. Introduction

Endothelial-overexpressed lipopolysaccharide-associated factor 1 (*EOLA1*, GenBank number 074889, also named as* CXorf40A*) has been identified by our research group from the genes with differential expression after stimulation of ECV304 cells with lipopolysaccharide (LPS) in 2004 [1, 2]; however its biological function is less understood.* EOLA1* is located at human chromosome Xq28 and contains 6,248 bases in genomics and 5 exons separated by 4 introns. The* EOLA1* gene is highly conserved in chimpanzee, rhesus monkey, dog, mouse, rat, chicken, zebrafish, and frog. EOLA1 protein comprises 158 amino acids and has several glycosylation and phosphorylation sites and the helix-turn-helix motif without signal peptide and membrane spanning domain. These structural features indicate that EOLA1 is an intracellular protein and may interact with other unknown proteins in the cytoplasm and nucleus to regulate cell function. The bioinformatics analysis reviewed that EOLA1 gene belongs to the activating signal cointegrator-1 (ASC-1) homology (ASCH) superfamily, which is frequently observed in many organisms [3, 4].

EOLA1 is weakly expressed under the steady condition and highly expressed in response to LPS in ECV304 cells. The discovery of the protein association between EOLA1 and metallothionein 2A (MT2A) has been confirmed by yeast double-hybridization and coimmunoprecipitation assay [2, 5]. MT2A is known to have anti-inflammatory, antiendotoxin, and tumor-inhibiting effects in cell cultures [6, 7]. In addition, MT2A inhibits cell growth through the induction of apoptosis and G2/M arrest, which negatively regulates NF-*κ*B pathway by upregulation of I*κ*B-*α* and downregulation of p-I*κ*B-*α* and cyclin D1 [8]. Based on a rat liver transplantation model, we have found the significant changes of EOLA1 expression in transplantation rejection [9]. These results suggest a possible role of EOLA1 in transmitting inflammation-related signals through association with MT2A and thus participating the regulation of inflammatory response in endothelial cells.

LPS is the major outer surface membrane component present in almost all Gram-negative bacteria and acts as ligand in triggering innate immune response via specific receptors in diverse eukaryotic species ranging from insects to humans [10, 11]. The induction of vascular cell adhesion molecule-1 (VCAM-1) is the hallmark of activated vascular endothelial cell [12]. VCAM-1 belongs to the immunoglobulin superfamily, regulates leukocyte recruitment and immune response to sites of inflammation, and accelerates the development of atherosclerosis [13, 14]. LPS is also known to induce inflammatory immune response at least in part via the upregulation of VCAM-1 expression in vascular endothelial cells.

To further understand the biological function of EOLA1 in regulating inflammatory immune response, we focused on determining the subcellular distribution of EOLA1 and its efficacy in the regulation of LPS-induced inflammatory gene response including VCAM-1 in ECV304 cells.

## 2. Methods

### 2.1. LPS Preparation and Cell Culture

Purified native LPS phenol extracted from* E. coli* serotype 0111: the B4 (Sigma) was dissolved in phosphate-buffered saline (PBS) to the dilution of 1 mg/mL and stored at −20°C. For experiments, the LPS stock solution was further diluted into supplemented tissue culture media to the working concentration of 100 ng/mL. ECV304 cells (ATCC) were cultured in Medium 199 supplemented with 10% fetal bovine serum, 3 mg/mL HEPES, 100 U/mL penicillin G, 100 mg/mL streptomycin, and 6 U/L insulin on gelatin coated dishes in a humidified atmosphere of 5% CO_2_. Confluent cultures were detached with EDTA-trypsin solution and split into 1 : 3 for further use.

### 2.2. Construction of EGFP-EOLA1 Fusion Protein Expression Plasmid and Cell Transfection

A pair of primers was synthesized according to the open reading frame (ORF) sequence of EOLA1 (forward primer: 5′-ACCAGGATCCTCTCTTCATGCCCAAAG-3′, reverse primer: 5′-AGCAGGATCCTCTCTTCATGCCCCAAAG-3′);* EcoR* I and* BamH* I endonuclease sites were introduced into the terminals of forward and reverse primers, respectively. ECV304 cells were stimulated with LPS (100 ng/mL) for 6 h, and then the total RNA was extracted for RT-PCR (TaKaRa), and finally the ORF sequence of EOLA1 was amplified. The PCR product and pEGFP-N2 plasmid (Clontech) were cut with endonucleases* EcoR* I and* BamH* I, then the enzyme cutting products were recovered by electrophoresis and linked with DNA ligase (TaKaRa), and finally the target ORF sequence was inserted into pEGFP-N2 plasmid and single colonies were selected for sequencing (TaKaRa) after transformation of competent* Escherichia coli* DH5*α*.

For cell transfection, ECV304 cells were split onto 6-well culture plate (precovered with a sterile coverslip) by 1.5 × 10^5^/well and continually cultured. Then, the fusion protein expression plasmid pEGFP-EOLA1 and the control plasmid pEGFP-N2 were transfected into cells using the liposome Lipofectamine 2000 (Invitrogen) for 48 h. The transfection efficiency was determined by GFP positive versus negative cells.

### 2.3. Subcellular Distribution of EOLA1

After cell transfection for 48 h, the culture medium was removed, and then the cells were rinsed twice with PBS and fixed with 4% PFA. A part of cells was used to examine the subcellular location; the cells were incubated with rat anti-GFP antibody (primary antibody) and goat anti-rat antibody (secondary antibody), stained with DAPI, and photographed under LSM510 META laser confocal microscope (Zeiss). For observation under immunoelectron microscope, the cells were fixed with 4% PFA and then oxidized with 3% hydrogen dioxide and incubated with serum. The incubation was performed with the previously prepared rabbit anti-EOLA1 polyclonal antibody as primary antibody and HRP-labeled goat anti-rabbit IgG antibody as secondary antibody. Then the cells were stained with HRP-labeled streptoantibiotin and diaminobenzidine (DAB), refixed with 2.5% glutaral and fixed with 0.1% osmic acid, dehydrated with acetone at a gradient, and then preserved overnight in the saturated uranyl acetate prepared with 70% acetone. The cells were further rinsed with pure acetone, soaked with the mixture of acetone and epoxy resin (v : v = 1 : 1), treated with pure embedding medium (without epoxy resin curing promoter DMP-30) and embedding medium (with DMP-30) at 40°C, and embedded with epoxy resin 618. Finally the cells were made into ultrathin sections, stained with lead, and observed and photographed under TECNA110 transmission electron microscope (Philips).

### 2.4. Knockdown Experiments

Knockdown of EOLA1 was accomplished using RNA interference (RNAi). The oligonucleotide sequences of EOLA1 shRNA (small hairpin RNA) were as follows: sense: 5′-AGGAGGCAAGGATGTATTCTTCAAGAGAGAATACATCCTTGTTTTTTT-3′ and antisense: 5′-AATTAAAAAAAGGAGGCAAGGATGTATTCTCTCTTGAAGAATACATCCTTGCCTCCTGGCC-3′. For knockdown of MT2A, the oligonucleotide sequences of MT2A-shRNA were as follows: sense: 5′-GATCCGCCGTG ACCGTTTGCTATATTTCAAGAGAATATAGCAAACGGTCACGGTTTTTTGGAAA-3′ and antisense: 5′-AGCTTTCCAAAAAACCGTGACCGTTTGCTATATTCTCTTGAAATATAGCAAACGGTCACGGCG-3′. The shRNA expression cassette driven by H1 promoter was engineered by inserting those oligonucleotides into the plasmid pSilencer 3.1/H1 hygro (Ambion). An unengineered plasmid, pH1, was used as a blank control. The cells were transfected with pH1, pH1-EOLA1 shRNA, or pH1-MT2A shRNA with Lipofectamine 2000 in serum-free OPTI-MEMI (Invitrogen) according to the instruction manual. The concentration of DNA vectors for transfection was 8 *μ*g/mL in a final volume of 500 *μ*L. The cells were washed after 4 h incubation and resuspended in complete growth medium with serum for further experiments.

### 2.5. RT-PCR and VCAM-1 Assay

The total RNA was isolated from the cells using TRIzol reagent following the manufacturer's instructions (Invitrogen). To measure the gene mRNA expression level, RT-PCR was performed using one-step reagent kit (TaKaRa). The primers are synthesized from commensal (EOLA1 upstream: 5′-GCTCGAATTCATGAAGTTTGGCTGCCTCTC-3′, downstream: 5′-AGCAGGATCCTCTCTTCATGCCCCAAAG-3′; *β*-actin: upstream: 5′-CGGGAAATCGTGCGTGACATT-3′, downstream: 5′-CTAGAAGCATTTGCGGTGGAC-3′). For VCAM-1 assay, the cells were homogenized in PBS (1 : 2, w/v) containing 1% protease inhibitors and then centrifuged at 12,000 ×g for 20 min at 4°C. The anti-EOLA1 polyclonal antibody was produced from New Zealand white rabbits immunization and identified by Western blot. IgGs against EOLA1 were purified on a protein A Sepharose column. The efficiency of polyclonal antibody was measured by ELISA analysis. The lysates of ECV304 cells were analyzed by Western blotting with antibodies to EOLA1. The supernatants were analyzed using ELISA kit (Sigma) according to the manufacturer's instructions.

### 2.6. Western Blot Analysis

Transfected cells were lysed with RIPA buffer supplemented with Complete Protease Inhibitor Tablets (Roche). Lysate protein concentration was measured by Lowry assay. The protein samples (30 *μ*g) were loaded on SDS-9% polyacrylamide gels and then transferred to Protan nitrocellulose membranes in an electroblotting apparatus, using standard procedures. Immunodetection was performed as described previously [2] by using anti-EOLA1, anti-MT2A, or anti-GFP as primary antibody and secondary antibody Ig-horseradish peroxidase conjugate (Invitrogen), anti-*β*-actin as loading control. The anti-EOLA1 polyclonal antibody was produced from New Zealand white rabbits immunization and identified by Western blot. IgG against EOLA1 was purified on a protein A Sepharose column. The efficiency of polyclonal antibody was measured by ELISA analysis.

### 2.7. Statistical Analysis

Data were expressed as means ± standard error. Student's *t*-test was performed to determine the statistical significance for the assays of ELISA; *p* < 0.05 was considered statistically significant.

## 3. Results

### 3.1. Subcellular Distribution of EOLA1 in ECV304 Cells

**Figure 1:**
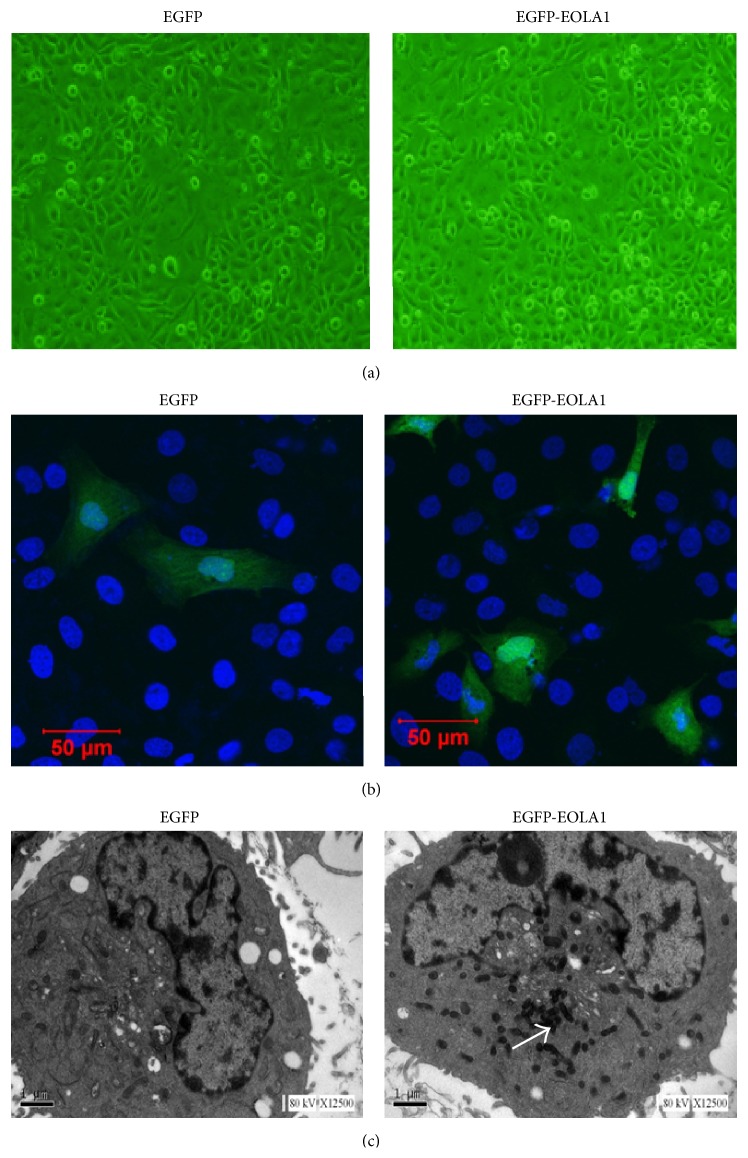
Subcellular location of EOLA1 is indicated in ECV304 cells. ECV304 cells were transfected with the blank plasmid pEGFP-N2 and the fusion protein expression plasmid pEGFP-EOLA1. After 48 h culture, the transfected cells were fixed with 4% PFA. (a) The images of ECV304 cells were taken under the optical microscope. (b) The cells were incubated with rat anti-GFP antibody (primary antibody) and goat anti-rat antibody (secondary antibody), stained with DAPI, and photographed under LSM510 META laser confocal microscope. (c) The cells were incubated with rabbit anti-EOLA1 polyclonal antibody (primary antibody) and HRP-labeled goat anti-rabbit IgG antibody (secondary antibody), stained with HRP-labeled streptoantibiotin and diaminobenzidine, and embedded with epoxy resin 618. Finally the cells were made into ultrathin sections, stained with lead, and observed and photographed under TECNA110 transmission electron microscope. The white arrow identifies immune sediment deposition in ECV304 cells transfected with pEGFP-EOLA1 by anti-EOLA1 antibody.

ECV304 cells were transfected with pEGFP-EOLA1 fusion protein expression plasmid and the control of pEGFP-N2 plasmid for 48 h. The images were observed under laser confocal microscope. ECV304 cells transfected with either control plasmid or fusion protein expression plasmid demonstrated uniform whole-cell distribution by green fluorescence, while the cells transfected with fusion protein expression plasmid also had nuclear aggregation (Figures 1(a) and 1(b)). With the prepared rabbit anti-EOLA1 polyclonal antibody as primary antibody, the transfected cells were labeled immunologically and then observed under electron microscope. There was no immunological precipitate in the cells transfected with control plasmid, but significantly immunological precipitates were deposited in the cytoplasmic ground substance of cells transfected with fusion protein expression plasmid (Figure 1(c)).

### 3.2. LPS Induction of EOLA1 Expression and VCAM-1 Production in ECV304 Cells

**Figure 2:**
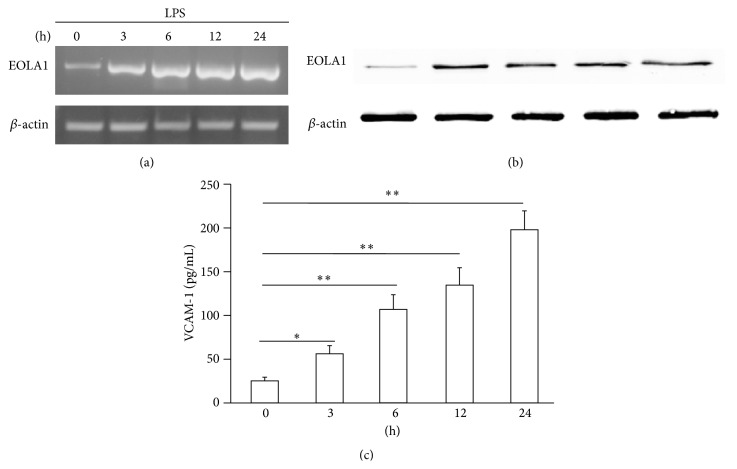
LPS induces the production of EOLA1 and VCAM-1 in ECV304 cells. (a) ECV304 cells were treated with LPS (100 ng/mL) for various periods of time, 0, 3, 6, 12, and 24 h, and the total RNA was analyzed by RT-PCR to EOLA1. (b) The lysates of ECV304 cells were analyzed by Western blotting with antibodies to EOLA1. Data show one representative experiment out of three independent experiments. (c) ECV304 cells were stimulated with LPS (100 ng/mL) for the indicated periods of time and the supernatants were collected and assayed for VCAM-1 production by ELISA. Data represent the mean ± SD of three independent experiments.

ECV304 cells were grown to 80% confluence and then stimulated with LPS (100 ng/mL) for a time-course as indicated. The cells showed that EOLA1 expression was increased in a time-dependent manner at both gene transcription (Figure 2(a)) and translation (Figure 2(b)). VCAM-1 concentration in the clarified culture supernatant was then measured by ELISA. Exposure of the cells to LPS (100 ng/mL) resulted in significant increase of VCAM-1 production after 3 h (*p* < 0.05) (Figure 2(c)). All the data were repeated by three independent experiments.

### 3.3. Overexpression of EOLA1 Inhibited the Expression of VCAM-1 Induced by LPS in ECV304 Cells

**Figure 3:**
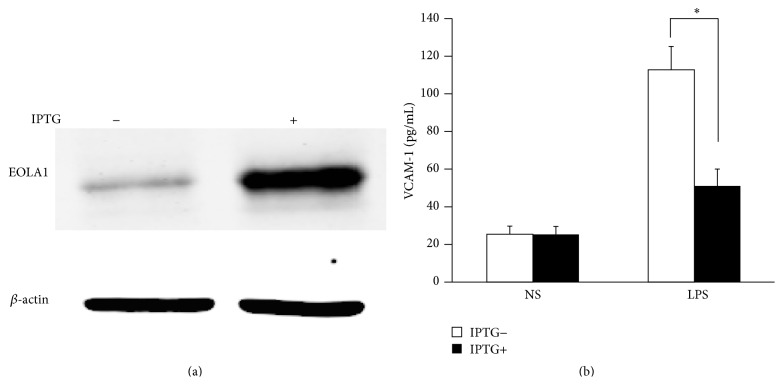
Overexpression of EOLA1 prevents LPS-induced VCAM-1 production in ECV304 cells. ECV304 cells were transfected with pOPRSVI-EOLA1-EGFP and pCMV-LacI plasmids and induced by IPTG. (a) Overexpression of EOLA1 was demonstrated by Western blotting analysis with anti-EOLA1 antibody after adding IPTG for 48 h. (b) The induced cells with IPTG were tested for their ability to produce VCAM-1 upon LPS stimulation (100 ng/mL) for 6 h using a commercial ELISA kit. Data represent the mean ± SD of three independent experiments (^*∗*^ *p* < 0.05).

To further characterize the functional effects of EOLA1 on VCAM-1 induction in ECV304 cells, the cells were cotransfected with pOPRSVI-EOLA1-EGFP and pCMV-LacI plasmids to stably overexpress EOLA1 after induction by IPTG (Figure 3(a)). The cells with overexpression of EOLA1 were treated with LPS (100 ng/mL) after adding IPTG for 48 h. The VCAM-1 protein production in cell supernatant was detected by ELISA assay in 6 h after LPS treatment. The results show that LPS significantly induces VCAM-1 protein secretion, and overexpression of EOLA1 reduces the VCAM-1 protein level induced by LPS in ECV304 cells (Figure 3(b)).

### 3.4. Knockdown EOLA1 Enhances LPS-Induced VCAM-1 Production in ECV304 Cells

**Figure 4:**
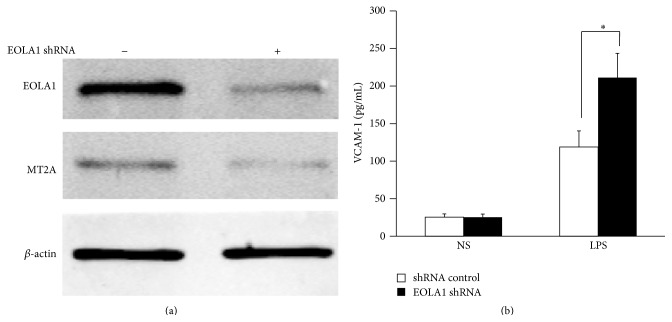
Knockdown of EOLA1 with shRNA inhibits the expression of MT2A and increases the LPS-induced VCAM-1 production in ECV304 cells. (a) ECV304 cells were transfected with control or EOLA1 shRNA for 48 h. The cells were lysed and analyzed by Western blotting with anti-EOLA1 or anti-MT2A antibodies, *β*-actin as a loading control. (b) ECV304 cells were transfected with control or EOLA1 shRNA for 24 h and then cultured with or without LPS (100 ng/mL) and the concentration of VCAM-1 in the culture supernatants was determined 6 h after stimulation. Data represent the mean ± SD of three independent experiments (^*∗*^ *p* < 0.05).

To further investigate the efficacy of EOLA1 on LPS-induced VCAM-1 production, the ECV304 cells were transfected with EOLA1 shRNA to silence EOLA1 gene. A significant downregulation of EOLA1 expression was confirmed in EOLA1 shRNA transfected cells by Western blot. Knockdown EOLA1 also showed the reduction of MT2A expression (Figure 4(a)). Knockdown EOLA1 resulted in the enhancement of LPS-induced VCAM-1 protein secretion (Figure 4(b)).

### 3.5. EOLA1 Regulates VCAM-1 Production through MT2A

**Figure 5:**
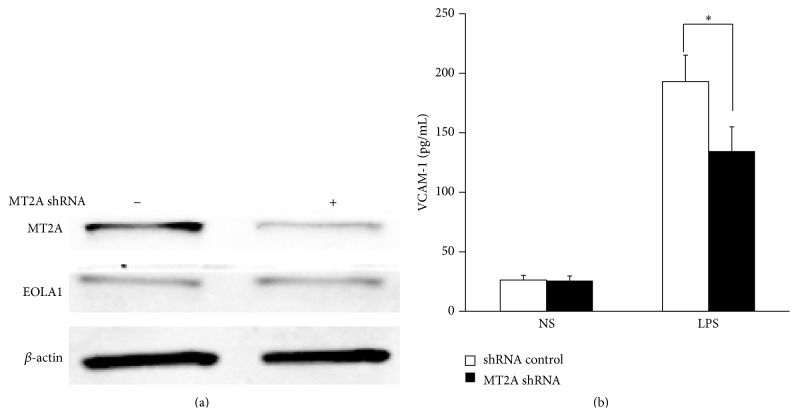
EOLA1 mediates LPS-induced VCAM-1 production by MT2A. (a) ECV304 cells were transfected with MT2A shRNA or control shRNA, and the cells were lysed and analyzed by Western blotting with anti-MT2A or ant-EOLA1 antibody, *β*-actin as a loading control. (b) ECV304 was transfected with MT2A shRNA or control shRNA and then stimulated with LPS (100 ng/mL) for 6 h. VCAM-1 production was assayed by ELISA. Data represent the mean ± SD of three independent experiments (^*∗*^ *p* < 0.05).

EOLA1 has an association with MT2A in ECV304 cells, and EOLA1 regulates the expression of MT2A, suggesting EOLA1 mediates VCAM-1 production through association with MT2A. We further investigated the role of MT2A in LPS-induced immune response. MT2A knockdown cells were treated with LPS, and the cell supernatant of VCAM-1 was measured by ELISA assay in 6 h after LPS treatment. The results show that knockdown MT2A downregulates LPS-induced VCAM-1 protein secretion in ECV304 cells (Figure 5).

## 4. Discussion

*EOLA1* is a novel gene firstly identified by our research group from the screening of LPS-induced gene expression in ECV304 cells; however, little is known about its biological function in inflammation. To understand the subcellular distribution of EOLA1, we directionally cloned the ORF sequence of EOLA1 into pEGFP-N2 carrier and thus successfully constructed the eukaryotic expression plasmid of EGFP-EOLA1 fusion protein to transfect ECV304 cells. Furthermore, we observed the transfected cells under laser confocal microscope after transient expression. The observations showed strong green fluorescence signals in both the cytoplasm and nucleus. The DAPI staining confirmed that the fusion protein was expressed in the cytoplasm and aggregated in the nucleus. Besides, we further observed the subcellular localization of EOLA1 at a level of cell ultrastructure by the use of immunoelectron microscopic enzyme labeling technique, and the results confirm that the expression of EOLA1 is mostly localized in the cytoplasm. The above observations indicate that EOLA1 is an intracellular protein and has a capacity to translocate into the nucleus from the cytoplasm, although such phenomenon still needs to be further confirmed in the future.

ECV304 cells belong to human umbilical vein endothelial cells, which can be activated by LPS stimulation. LPS has been shown to initiate TLR4 mediated intracellular signaling events, including the activation of NF-*κ*B, which ultimately leads to the transcription and release of VCAM-1 in endothelial cells to attract mononuclear macrophage accumulation for inflammatory response [15, 16]. Recent study also showed that LPS-induced VCAM-1 expression contributes to the initiation of the atherosclerotic process [17, 18]. Our experiments showed that LPS induces EOLA1 expression along with VCAM-1 induction, overexpression of EOLA1 reduces the LPS-induced production of VCAM-1 in ECV304 cells, and depletion of EOLA1 significantly enhances the LPS-induced production of VCAM-1. Our results suggest that EOLA1 regulates the degree of endothelial cell activation by LPS and possibly negatively regulates the inflammatory immune response.

MT2A is a low-molecular-weight protein in the cytoplasm, plays multiple roles in cell metabolism, and has the effects on anti-inflammation and antiendotoxin and inhibiting tumor growth. Recent report indicates that MT2A reduces cell inflammatory response by inhibiting the activation of NF-*κ*B, through upregulating I*κ*B-*α*. Our results show that EOLA1 regulates the expression of MT2A and affects the production of VCAM-1 by association with MT2A; therefore the excessive activation of ECV304 cells could be avoided, and the fine regulation and timely control of inflammatory reaction could be thereby achieved. This will avoid the damage of excessive inflammatory reaction to tissues and cells. The detailed mechanism of EOLA1 association with MT2A and its functional effects on the NF-*κ*B signaling pathway needs to be further characterized.

In conclusion, EOLA1 plays a negative regulatory role in LPS-induced VCAM-1 expression under the association with MT2A in ECV304 cells.
