# Corrigendum to “EOLA1 Inhibits Lipopolysaccharide-Induced Vascular Cell Adhesion Molecule-1 Expression by Association with MT2A in ECV304 Cells”

**DOI:** 10.1155/2020/3503814

**Published:** 2020-09-27

**Authors:** Weiling Leng, Xiaotian Lei, Hao Meng, Xinshou Ouyang, Ziwen Liang

**Affiliations:** ^1^Department of Endocrinology, Southwest Hospital of Third Military Medical University, Chongqing, China; ^2^Section of Digestive Diseases, Department of Internal Medicine, Yale University School of Medicine, New Haven, CT, USA

In the article titled “EOLA1 inhibits lipopolysaccharide-induced vascular cell adhesion molecule-1 expression by association with MT2A in ECV304 cells” [1], the ECV-304 cell line has been found to be cross-contaminated by T-24, which is a bladder carcinoma cell line [2, 3].
